# Prescription drug abuse in migrants from Middle Eastern and North African countries: a review

**DOI:** 10.1192/j.eurpsy.2023.1368

**Published:** 2023-07-19

**Authors:** C. Portela, C. Oliveira, M. Gonçalves

**Affiliations:** Hospital de Magalhães Lemos, Porto, Portugal

## Abstract

**Introduction:**

In recent years, there has been a rise in misuse of low-cost prescription pills across Middle Eastern and North African (MENA) countries. In Algeria, Tunisia and Morocco, for example, the consumption of prescription medications has dramatically increased, particularly amongst young and marginalized groups. Drugs such as clonazepam and pregabalin are extremely popular in these regions, as they are relatively inexpensive and perceived as safe. With the migration of MENA citizens to Europe, it is likely that mental health services will come across substance use disorders related to these medications.

**Objectives:**

The authors aim to analyse prescription medication misuse reports from MENA countries, specifically pregabalin and clonazepam, and review the pharmacological, neurobiological and social factors that contribute to their potential for abuse.

**Methods:**

Narrative review of articles referenced on PubMed and Google Scholar.

**Results:**

Pregabalin and clonazepam are widely used in psychiatry and neurology. Pregabalin is an alpha 2 omega ligand with supposed GABA-mimetic properties. Anecdotal reports suggest that pregabalin, used recreationally in amounts up to 3-20 times the therapeutic doses, possesses both sedative and psychedelic effects. Experimenters are mainly individuals with a history of recreational polydrug use, who are aware that pregabalin is not included in standard drug monitoring tests, with this molecule being used in some instances as a legal substitute of common illegal drugs. Clonazepam is a benzodiazepine that combines high potency and a long duration of action and is said to cause euphoria at doses over 8mg. It is very popular and affordable, placing consistently in the top three of benzodiazepines sales across the globe. Clonazepam has potential for tolerance build up and severe withdrawal symptoms. These medications are frequently used together and in combination with other substances such as alcohol and opiates, increasing the risk for respiratory failure and death.

**Conclusions:**

Prescription medications such as pregabalin and clonazepam are extremely accessible, inexpensive and highly addictive substances, whose abuse is well disseminated across MENA countries. With migratory flows from this region, the prevalence of misuse of these drugs in Europe is expected to increase. Therefore, physicians should be aware of their potential for abuse and carefully evaluate patients’ previous history before prescribing these medications.

**Disclosure of Interest:**

None Declared

